# Back to basics in COVID‐19: Antigens and antibodies—Completing the puzzle

**DOI:** 10.1111/jcmm.16462

**Published:** 2021-03-18

**Authors:** Monica Neagu, Daniela Calina, Anca Oana Docea, Carolina Constantin, Tommaso Filippini, Marco Vinceti, Nikolaos Drakoulis, Konstantinos Poulas, Taxiarchis Konstantinos Nikolouzakis, Demetrios A. Spandidos, Aristidis Tsatsakis

**Affiliations:** ^1^ Department of Immunology Victor Babes National Institute of Pathology Bucharest Romania; ^2^ Department of Pathology Colentina Clinical Hospital Bucharest Romania; ^3^ Doctoral School University of Bucharest Bucharest Romania; ^4^ Department of Clinical Pharmacy University of Medicine and Pharmacy of Craiova Craiova Romania; ^5^ Department of Toxicology University of Medicine and Pharmacy of Craiova Craiova Romania; ^6^ Section of Public Health Department of Biomedical, Metabolic and Neural Sciences Environmental, Genetic and Nutritional Epidemiology Research Center (CREAGEN) University of Modena and Reggio Emilia Modena Italy; ^7^ Department of Epidemiology Boston University School of Public Health Boston MA USA; ^8^ Research Group of Clinical Pharmacology and Pharmacogenomics Faculty of Phrarmacy School of Health Sciences National and Kapodistrian University of Athens Athens Greece; ^9^ Department of Pharmacy Laboratory of Molecular Biology and Immunology University of Patras Patras Greece; ^10^ Department of Forensic Sciences and Toxicology Faculty of Medicine University of Crete Heraklion Greece; ^11^ Laboratory of Clinical Virology Medical School University of Crete Heraklion Greece; ^12^ Department of Analytical and Forensic Medical Toxicology Sechenov University Moscow Russia

**Keywords:** antibodies, antigens, immune memory, immune response, mutations, SARS‐CoV‐2, tests

## Abstract

The outbreak of the coronavirus disease 2019 (COVID‐19) has gathered 1 year of scientific/clinical information. This informational asset should be thoroughly and wisely used in the coming year colliding in a global task force to control this infection. Epidemiology of this infection shows that the available estimates of SARS‐CoV‐2 infection prevalence largely depended on the availability of molecular testing and the extent of tested population. Within molecular diagnosis, the viability and infectiousness of the virus in the tested samples should be further investigated. Moreover, SARS‐CoV‐2 has a genetic normal evolution that is a dynamic process. The immune system participates to the counterattack of the viral infection by pathogen elimination, cellular homoeostasis, tissue repair and generation of memory cells that would be reactivated upon a second encounter with the same virus. In all these stages, we still have knowledge to be gathered regarding antibody persistence, protective effects and immunological memory. Moreover, information regarding the intense pro‐inflammatory action in severe cases still lacks and this is important in stratifying patients for difficult to treat cases. Without being exhaustive, the review will cover these important issues to be acknowledged to further advance in the battle against the current pandemia.

## 1 INTRODUCTION

The pandemic that we are facing has reached its first year, registering on 18th of February 2021, over 109 million COVID‐19‐confirmed cases worldwide and over 2.4 million death; the confirmed cases being mainly in Americas (almost 49 million cases) and Europe (over 37 million cases) as reported to the WHO.^1^

The SARS‐CoV‐2 pandemic has changed in science so many things. It has speeded research in virus identification, in therapy, in epidemiology, and even in our scientific language. The word ‘recently’ that is commonly used in publication has a different time span now. As for another topics ‘recently’ would mean the previous year or this year, now ‘recent’ means ‘this month or even these days’. But this unprecedent speed in science comes with a toll. As the infectious agent is somewhat new, there is still a puzzle consisting of various information that needs to be completed in several areas. Therefore, in the epidemiological domain the various mortality rates in different geographical areas vary. This variation has still unknown cause, probably related to age, to comorbidities or other susceptibility factors. Another important issue is the genetic variability of the SARS‐CoV‐2 variant that favoured the spill‐over between species. The ongoing mutational mechanisms favour its infectivity but the association with aggressivity is still unknown. Mutation frequency controls the establishment of a proper (immune) therapy. In this sense, a therapy that was specific to one variant may be of reduced efficacy in a mutated one. Last, but not the least, it is still unknown if a populational immunity established naturally or artificially through vaccination can offer the same protection for a continuously mutating variant. All these issues will be addressed in the paper.

## 2 EPIDEMIOLOGY

The epidemiology of SARS‐CoV‐2 infection and its related disease COVID‐19 in the human has been extensively investigated all over the world, with reference to its incidence over time and space, the related risk factors, and the potentially effective therapy.^2^, ^3^ In addition to this and based on the large amount of epidemiologic evidence available on this infection and the disease, sophisticated predictive models have also been developed, aiming at anticipating the subsequent waves of the outbreak and the impact of public health measures in curbing it.^4^, ^5^

The SARS‐CoV‐2 infection in the human was first identified in China (Wuhan, Hubei Province) on 8 December 2019.^6^ It swept outside China in early 2020, and Italy was the first country, both in Europe and worldwide, to be severely hit by the epidemic, which swiftly spread across this country in early March following the detection of the index case on February 21 in the Codogno Hospital, Lombardy region. However, evidence that the virus was present in Europe, namely in Italy^7^ and in France,^8^ already in December 2019 has been recently provided, giving the possibility to advance the beginning of the outbreak 3 months before the first Italian reported case of non‐imported origin. The WHO declared the COVID‐19 outbreak to be a pandemic on 11 March 2020, with so far 70 461 926 cases of SARS‐CoV‐2 infections and 1 599 704 deaths (WHO data base, 2020). Nowadays, the number of diagnosed SARS‐CoV‐2 infections and its death toll is still quickly increasing across most countries of the world (GIS‐JH),^9^ though with a rather uneven geographical distribution. In fact, incidence, mortality and lethality of COVID‐19 greatly varied across countries and continents, due to unknown factors and to some known determinants such as older age, gender and presence of comorbidities such as chronic diseases. Both age and comorbidities are independently associated with a high susceptibility to the infection and its clinical serious effects.

Infection with SARS‐CoV‐2 mainly occurs following airborne transmission due to droplets and aerosols, other much more unlikely possibilities being contact with infected surfaces.^10^ Food intakes are not considered to be a source of infection.^11^ Although there is evidence that the infectious dose of SARS‐CoV‐2 is lower compared with other airborne viral diseases such as influenza,^12^ on the contrary, it appears to be higher than other extremely contagious viral diseases such as Q fever and measles,^13^ possibly explaining why limited interactions with infected individuals may not be enough to transmit the infection itself.^14^ Closed and crowded environments or some outdoor settings can favour transmission.^10^, ^15^ This has led to the adoption of public health measures to prevent the infection such as mobility restrictions (lockdowns), recommendations for social distancing and use of hand sanitizer with alcohol‐based formulation, along with use of personal protective equipment such as disposable gloves and face masks. These measures turned out to be highly effective in curbing the outbreak^16^ though at the expense (for lockdowns) of huge economic and psychological consequences.^17^ Additional factors may favour the spread of the infection and related clinical manifestations, including air and environmental pollution^18^, ^19^, ^20^ possibly through a weakening of the immunological response and an increased prevalence of chronic diseases, and meteorological factors such as humidity and temperature (apparently the higher the better to counteract SARS‐CoV‐2 diffusion, though convincing evidence on this is still missing).^21^, ^22^ Although SARS‐CoV‐2 infection may well be asymptomatic through its entire course, in many cases it leads to the onset of mild to severe clinical symptoms such as fever, cough, and particularly interstitial and potentially extremely severe pneumonia, that is COVID‐19.^23^ However, both symptomatic and asymptomatic SARS‐CoV‐2‐infected individuals may transmit the pathogen.^24^

Several studies reported that COVID‐19 incubation period is generally 5‐6 days (median 5.1, 95% CI 4.5‐5.8 days), with 97.5% of subjects developing symptoms within 11.5 days.^25^, ^26^ Nonetheless, the incubation may widely range, from 2 to 15 days, and a shorter incubation period appears to be associated with severe progression and aggravation of pneumonia.^27^

Although here is no doubt about the high lethality of COVID‐19, much higher than that of seasonal flu, the case‐fatality rate is still not well defined since it varies across different countries, also depending on risk factors such as older age and comorbidities, namely respiratory, renal and cardiovascular disease.^28^ Even more uncertain and complex is the identification of infection fatality rate, since the actual prevalence of the SARS‐CoV‐2 infection is quite difficult to determine, depending on the availability of molecular testing and the extent to which the population or selected subgroups are systematically tested.^29^ For instance, different country‐specific testing strategies may at least partially explain the uneven distribution of COVID‐19 fatality rate across the world, due to the limited availability of tests, especially during the first months of the pandemics. During the first wave of COVID‐19, in fact, countries such as Italy prioritized testing for patients with severe clinical symptoms who were suspected of being infected and required hospitalization, resulting in an extremely high but potentially misleading proportion of fatal cases.^30^

## 3 ANTIGENS

### 3.1 Strains similarities with prior viral infections

#### 3.1.1 From bats to humans

The available scientific data suggest that SARS‐CoV‐2 virus has a natural origin, transmitted from animals. In fact, most of the major viruses that we are facing in the last century are zoonotic. Therefore, Ebola outbreaks have started in bats,^31^ and primates,^32^ Zika from primates *via* mosquitoes,^33^ HIV is derived from the SIV virus known in chimpanzees and gorillas,^34^ and bird and swine flu have an obvious source. The two related beta‐coronaviruses, SARS‐CoV and MERS‐CoV, are also zoonotic, having as primary origin bats. If SARS appears to have been transmitted to humans through civets sold at animal fairs in China, MERS has as its secondary host the dromedaries.^35^ The viruses have adapted to the selection pressure of the environment, and the main changes are frequent mutations in the genome called genetic drift, mutations that are both cumulative and random. These mutations can be favourable for the virus, helping the variant to proliferate to the detriment of less competitive strains; meanwhile, other mutations are unfavourable and would lead to extinction.^36^

Genetic drift mutation rates are a variable parameter among viruses, but RNA viruses (such as SARS‐CoV‐2) mutate more often than DNA viruses, and the mutation rate may increase when selection pressure is high.^36^ Influenza virus genetic drift that would generate a different antibody pattern has driven flu vaccines to be annually updated.^37^ Viruses have other, more abrupt adaptation strategy; namely, they can change a larger part of the genome, increasing their infectivity or even ‘jumping’ from one species to another. In coronaviruses, this genetic recombination can be triggered by the coexistence of two or more virus strains in the same host; therefore, the strains can ‘mix’ their genes to form a hybrid virus.^38^ Relative immediately after the identification of COVID‐19 as a new type of acute respiratory syndrome,^20^, ^23^ a complete sequencing of the viral genome was done, and once the RNA genetic fingerprint was known, comprising around 30 000 bases, comparisons could be made with known viruses to establish phylogeny, species spill‐over in time and space.^39^ During the same time, Chinese researchers identified the most similar sequence to the current SARS‐CoV‐2 in a new bat virus, called RaTG13, which is 96.2% identical to the virus responsible for COVID‐19, significantly more related than any previous candidate. It is so closely related that, together with SARS‐CoV‐2, it forms a separate subfamily of beta‐coronaviruses.^40^

‘Jumping’ from one species to another, this type of viruses opens new areas of both fundamental and applicative researches.

### 3.2 Mutation frequencies in SARS‐CoV‐2

The fact that SARS‐CoV‐2 has 80% identity with SARS‐CoV and 50% with MERS‐CoV, probably suggests a possible common origin. MERS‐CoV uses a transmembrane dipeptidylpeptidase 4 (DPP4) to infect cells, whereas SARS‐CoV and SARS‐CoV‐2 use angiotensin‐converting enzyme 2 as receptor (ACE2).^41^ Therefore, it is probable that they have a similar origin from different coronaviruses infecting bats.^42^ Mutations are taking place in key genes that encode for main proteins, like the receptor‐binding domain of S protein; mutations improving its binding to ACE2 receptor and hence increasing cell entrance.^43^

Mutational frequency of the virus increases, and these genetic mechanisms associate with an increase in the infection rate in the United States. Under positive mutational pressure, mutation frequency is higher in several proteins (eg NSP2, NSP3, RdRp, helicase, S, ORF3a, ORF8, N). The maximum mutations were detected in ORF8 and helicase.^44^ Mutations in these proteins sustain viral adaptation to human host and contribute to virulence and transmission, during the epidemic.^45^ The virus accumulates specific mutations in various geographical regions, Asia, Oceania, Europe and North America.^46^ Mutations occur as intrinsic viral mechanisms, as virus adaptation to new bio‐environment. The specificity of the new environment comprises population's biological characteristics, but also social characteristics, like access to healthcare system and/or other socio‐economic factors.^44^ Another recent study embarked in the comparative genomic analysis of over 80 SARS‐CoV‐2 genomes isolated from different regions of the world. Out of all the tested genomes, there were found four viral strains under purifying selection, and nine genomes under strong positive selection. The strong positive selection pressure was identified at 3606th and 8439th codon positions. The nine SARS‐CoV‐2 strains that are under strong positive selection came from Brazil, Australia, India, and the United States. The codons that are evolving under strong positive selection encode 3C‐like proteinase and spike protein.^47^ Another study that analysed mutation rate in four different regions: China, Australia, the United States, and the rest of the World has shown that nucleotides T and A mutate to other nucleotides. This study showed that approximately 0.1% increase in mutation rate was found for mutating T to C and G, C to G and G to T; a decrease is also seen for T to mutating to A, and A to C with the same 0.1%.^48^ In a press release done on the 19th of December by Dr Jeremy Farrar, Director of Wellcome, it was stated that a new variant of SARS‐CoV‐2 was detected in UK and this new variant is more transmissible increasing the R0 of the transmission. Concerns were raised that the vaccines that were so rapidly approved by FDA, EMA and UK would have a lower efficacy.^49^ To this recently raised concern, the extremely near future will show if this is a real concern or if the developed vaccines elicit cross‐reactivity with the new variant of virus. In terms of mutational frequency, the virus tries to increase its infectivity, inhibit host defence, and increase its inflammation‐related mechanism.

### 3.3 Immune memory

Classical immunological memory represents a faster and more efficient immune response in comparison to the primary immune response against the same pathogen. Memory T lymphocytes are developed, needing lower antigen concentration and lower co‐stimulatory signals, whereas B cells quickly proliferate and differentiate in antibody‐secreting plasma cells.^50^, ^51^, ^52^, ^53^, ^54^ Memory T cells when activated, at re‐challenge, will produce inflammatory mediators (eg IFN‐γ, CCL3, CCL4, CCL5), recruiting other immune cells. It is described that these cells may have a half‐life of 8 ‐ 15 years, surveying the immune response upon re‐challenge throughout an individual's life.^51^, ^52^, ^53^, ^55^ Memory B cells that are generated, return to the germinal centre, and they will activate, proliferate, and differentiate in plasma cells upon re‐challenge. These long‐lasting cells are capable of secreting cytokines upon activation.^56^

#### 3.3.1 Immune memory in coronavirus infection

The experience gained from the prior SARS infections has taught the medical world that specific humoral immunity like antibodies can be still detected; hence, IgG titres are detectable for more than 1‐year post‐infection.^57^ In addition, cellular immunity like T memory lymphocytes after severe SARS infection could be detected 6 years post‐infection.^58^, ^59^ Moreover, the hypothesis that antibodies generated during this infection could have some cross‐reactivity against SARS‐CoV‐2 can be raised due to several reasons. As memory cells recognize SARS‐CoV protein S that has similarities to SARS‐CoV‐2, there is an overlapping in the immune response, thus reducing symptoms for the new infection. A recent study demonstrated that there is indeed a cross reaction between antibodies against RBD and S1 regions for SARS‐CoV‐2 and SARS‐CoV patients but no cross‐neutralizing antibodies to SARS‐CoV‐2 and SARS‐CoV protein S.^60^

In MERS infections, antibodies at 13 months after infection are equivalent with the ones detected 3 years after the infection.^61^ However, in MERS infection tens of different antibodies were detected^62^ and probably this is one of the reasons why antibodies in SARS‐CoV‐2 infection are restricted to only few types and decrease 2‐3 months after infection.^63^

In experimental models it was shown that SARS‐CoV‐2 re‐infection in rhesus monkeys with the same variant, no viral replication was found and no symptomatology, demonstrating that neutralizing antibodies generated upon re‐infection stopped the infection.^64^

Another point to be considered is the specificity of the immunological memory. Re‐infected patients were reported but it is not clear if the re‐infection occurred with a different variant of the same virus or not.^65^, ^66^ Another study on seroprevalence dynamics has shown that seropositive samples were found as early as mid‐February, while from May to July, seroprevalence was stable, suggesting lasting antibody levels.^67^

As vaccines have entered, the road map for massive populational vaccination,^68^, ^69^, ^70^ in order to keep pace with the variants, genetic and antigenic surveillance is required.^71^ The experience gained for 70 years in influenza vaccines has shown that vaccines become ineffective as the virus rapidly mutates.^72^ In this last year from the moment, SARS‐CoV‐2 genome was first identified; the race for developing vaccines begun, but it remains to be elucidated whether and to what extent the capacity of vaccines can offer the same protections to all virus variants.^73^ The new variant has been detected in the UK where had already infected 1/4 of the total cases by December 2020. SARS‐CoV‐2 as well suffer many mutations that do not significantly modify the structure and the components of the virus; thus, mutations occured in SARS‐CoV‐2 as well. For now, it is probable that the UK variants should not hinder vaccine‐induced immunity, although it may lower its effectiveness. Therefore, for the very near future larger variants of the spike protein can occur so intense studies of spike protein mutations are needed.^74^, ^75^ Recent studies show that mRNA‐based approved vaccines elicit antibodies and B memory cells equivalent to the patients recovering from disease. The neutralizing capacity of the vaccine‐generated antibodies was slightly reduced when tested against new variants, pointing towards the fact that mRNA vaccines may need to be updated periodically to avoid potential loss of clinical efficacy.^76^ UK variant B.1.1.7 and South Africa variant B.1.351. B.1.1.7 were found modestly more resistant to convalescent plasma and vaccinee sera. But B.1.351 was found resistant to multiple individual mAbs to the receptor‐binding motif on RBD. This resistance is due to an E484K mutation. In comparison with the UK variant, B.1.351 is more resistant to neutralization by convalescent plasma and vaccinee sera.^77^

The immunological memory in this infection is of outmost importance as vaccination therapy stringently relies on it, but we still do not know if the vaccine will raise a sturdy immunological memory and how this memory will cover the emergence of new variants.

#### 3.3.2 SARS‐CoV‐2 immune responses

First immune response resides in the activation of macrophages and neutrophils, pro‐inflammatory cytokines that will activate NK cells with anti‐viral activity. If the virus still needs to be eliminated, the anti‐viral adaptive immune responses will reside on antigen‐specific CD8+ cytotoxic T cells (CTLs), Th1 subset of CD4+ T helper cells and plasma cells will secrete specific antibodies, and last, but not least, clones of T and B memory cells will be generated. A rapid immune response will lead to virus clearance and generation of immune memory. If the immune response is delayed due to various causes or due to comorbidities of the infected person, the viral infection cannot be abolished, and this could lead to poor clinical outcome and even death. For example, an inefficient virus clearance done by alveolar macrophages would lead to an enhanced viral replication.^78^, ^79^ On the contrary, individuals with robust immune responses, while not experiencing infection symptoms, can be silent spreaders of SARS‐CoV‐2.^80^ The experience gained from SARS‐CoV and MERS‐CoV infection has shown that a delayed type I IFN production and an excessive recruitment/activation of infiltrating neutrophils and monocytes‐macrophages (high pro‐inflammatory cells) are key mediators of lung dysfunctions. This delayed IFN response allows for intense viral replication and further recruitment of inflammatory neutrophils and monocytes that are further activated to secrete more pro‐inflammatory cytokines. All these immune‐related processes induce septic shock, lung failure, pneumonia or acute respiratory distress syndrome (ARDS).^81^ In SARS‐CoV‐2 infection, hyperactivated neutrophils and macrophages are found, whereas absolute neutrophil counts and neutrophil‐to‐lymphocyte ratio (NLR) were associated with worse outcome.^82^ Pro‐inflammatory cytokines detected in patient's serum were found associated with pulmonary inflammation and reported in the first 41 patients with COVID‐19 in Wuhan.^83^ ARDS cases proved high levels of cytokines and chemokines [tumour necrosis factor‐α (TNF‐α), IL‐2, IL‐6, IL‐8 and IL‐10]. Therefore, therapies that control these immune‐related effects such as Janus kinase (JAK) inhibition to reduce inflammation and viral entry^84^ or the use of corticosteroids, cytokine blockers, were in the clinical pipeline.^85^ Besides the rapid and immunologically ‘noisy’ innate immunity, the adaptive immunity would be triggered by virus presentation to T cell. SARS‐CoV and MERS‐CoV experience has shown that there are HLA alleles that can be found in susceptible to infection patients as well as HLA alles that offer protection,^86^ so probably in SARS‐CoV‐2, antigen presentation for specific HLA alleles could be found. Lymphopenia, CD8+ T lymphocytic cells decreased numbers, high naïve CD4+, low numbers of regulatory T cells (Tregs), manly induced Tregs are characteristics of worse outcome in COVID‐19 disease.^87^ Cross‐reactivity with SARS‐CoV antibodies and against other coronaviruses was found.^88^, ^89^, ^90^

Overall, SARS epidemic has shown that specific IgGs lasted for at least 2 years after infection. In anti‐SARS‐CoV‐2, antibodies high levels were not always neutralizing despite that critically ill patients could have high antibody titres.^91^ An interesting study has shown the existence of specific IgA.^92^ In terms of immunological memory persistence, we still need time to evaluate memory responses.

Antibody‐dependent enhancement (ADE) is a mechanism sustained by non‐protective antibodies that can facilitate virus entry in target cells^93^ and antibodies against different coronaviruses can induce ADE.^94^ In SARS‐CoV, the low affinity anti‐Spike protein antibodies activate the Fc receptor mediated infection^95^ hindering inflammation control mainly in the lung.^96^, ^97^ Although we still do not know if ADE can occur in SARS‐CoV‐2 infection,^98^ vaccine developed for this virus should bear in mind the Dengue vaccine story.^99^

**FIGURE 1:**
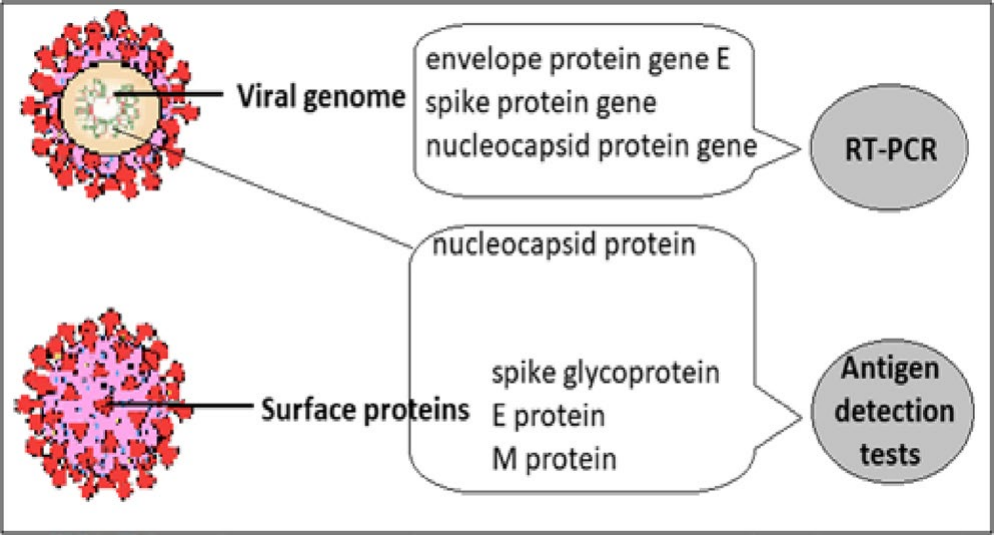
Main molecular targets and antigens detected in SARS‐CoV‐2 infection used in diagnosis

Tests that can be used to evaluate genome and proteome in this viral infection are presented in Table 1 and a schematic outline of the tests in Figure 1.

**TABLE 1:** Main caharacteristics of molecular and antigen tests in SARS‐CoV‐2

Test/characteristics	RT‐PCR	Antigen test
Detects	Viral genome	Proteins on the viral particle surface
Advantages	Accurate, identifies mutations in the virus, it tracks disease spred	Faster then molecular tests, less expensive, applicable to large number of samples
Disadvantages	Does not detect viral load, does not detect dynamics of infection or the history of prior infection	Less sensitive than molecular tests and often a molecular test needs to confirm the positive result
PPA (per cent positive agreement) sensitivity %	86.1%	61.7%
PNA (per cent negative agreement) specificity %	95.8%	98.2%

## 4 ANTIBODIES

### 4.1 Antibody dynamics in infection

In SARS‐CoV‐2 infection, IgM antibodies from the fourth day of infection, increasing until the 20th day (approximate peak), fading away while IgG appears from the seventh day, peaks on the twenty‐fifth day and maintains 1 month after infection.^100^ Seroconversion (the appearance of IgG or IgM antibodies) can take place simultaneously or sequentially, and after 6 days after seroconversion, the concentrations of the two types of antibodies reach a plateau value and no longer vary.^63^

In patients with mild and severe forms over time the IgM titres gradually increase.^101^ It was shown in the severe group compared to the non‐severe group that IgG and IgM titres are high. Patients with severe disease have a high IgG response but mild cases will develop a faster peak IgM response.^102^, ^103^

In asymptomatic or better oligo‐symptomatic patients, antibodies were detected but the titres are lower compared to symptomatic individuals. As other groups reported, we have also found that some of the oligo‐symptomatic patients became seronegative for IgG, in a high proportion 40.0% compared to only 12.9% in symptomatic patients.^104^

We should point out some details regarding antibodies and neutralizing antibodies. Neutralizing antibodies (NAbs) are the antibody populations that offer protection whether the infection is done naturally or artificially through vaccination. A NAb stops the pathogen from infecting the cells by hindering the mechanisms of viral entry.^105^ Moreover, NAbs impede conformational changes in the virus, changes that are related to the entrance in the target cell. This capacity of NAbs is used in passive immunization from convalescent plasma to patients that are still fighting the disease, and although it does not last like the own NAbs, it will offer an immediate protection.^106^ Another type of NAbs is the ones that block the receptors on the target cells, so that the virus cannot enter; although it is a neutralizing mechanism, it is named infection‐blocking mechanism. Several monoclonal antibodies against the spike protein of SARS‐CoV‐2 have been either isolated from convalescent plasmas or designed and further expressed de novo. This therapy is attempting to use the neutralizing antibodies to inhibit virus infection, but the results are not satisfactory yet.

**FIGURE 2:**
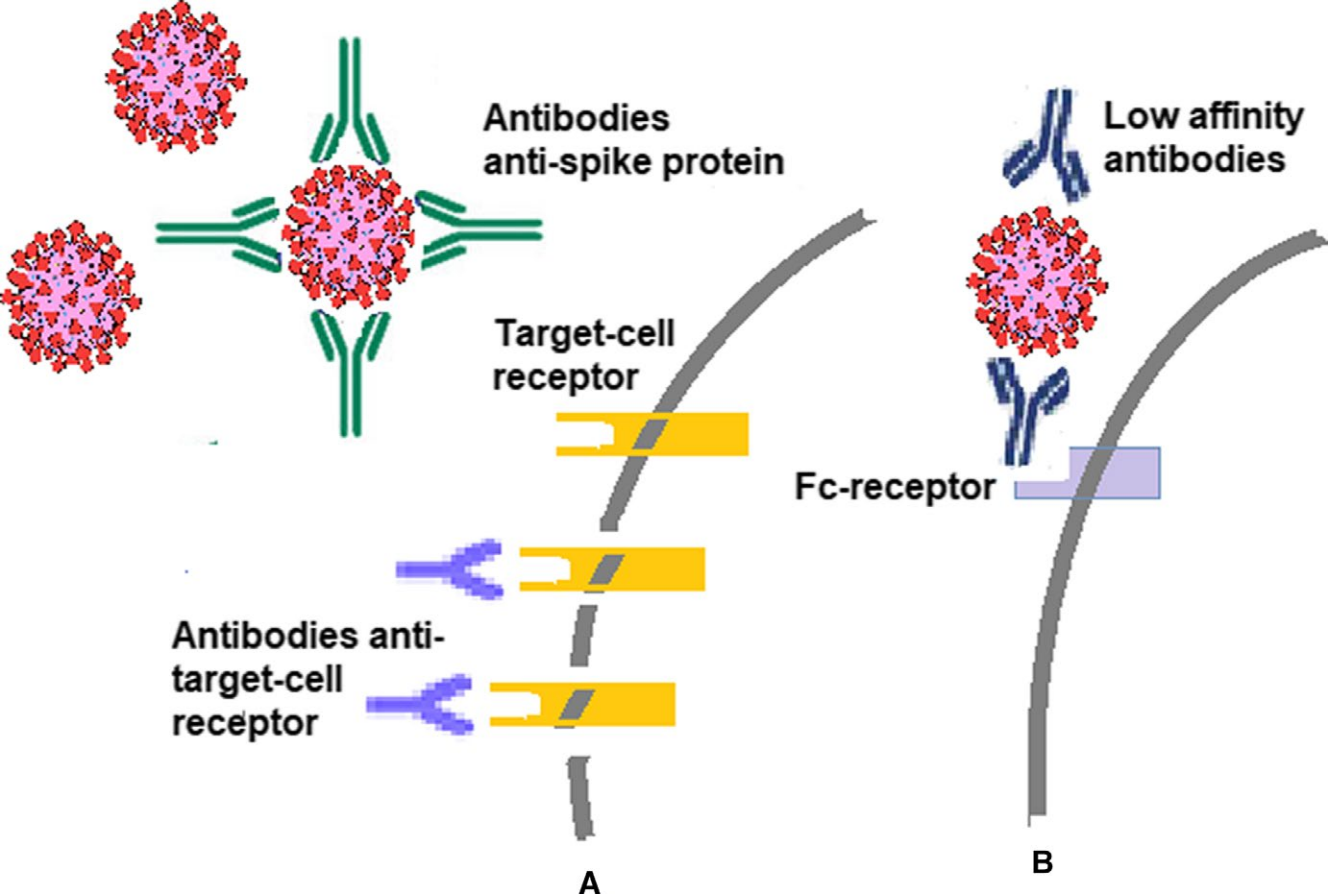
Antibody types in viral infections. (A) Neutralizing antibodies can link to the viural particle hindering its entrance in the target cell, and/or antibodies can link to the specific receptor that it used by the viral particle; (B) low affinity antibodies linked to the viral particle can activate Fc receptor on the target cell and thus favour viral entry into the cell generating ADE‐related mechanisms

Farsalinos et al^107^ have expressed the theory that SARS‐CoV‐2 is interacting with the nicotinic cholinergic pathway. Molecular modelling and docking experiments have proposed that there is a key interaction between the SARS‐CoV‐2 Spike glycoprotein and the nAChR alpha subunit extracellular domain (ECD). This interaction is mainly happening between the ‘cryptic epitope’ and the nAChRs' ‘toxin‐binding site’ (important aminoacids for this interaction are proposed to be within aa 365‐390).^108^ According to this theory, Spike glycoprotein is ‘toxifying’ and dysregulating the anti‐inflammatory cholinergic pathway. CR3022, COVA1‐16 and other overlapping Abs can be therapeutically useful for the inhibition of this interaction and not for the neutralization of the virus. Figure 2 depicts the schematic representation of NAbs and their mechanism; moreover, Abs that can lead to ADE in viral infection are represented as well.

## 5 DISCUSSION

### 5.1 A brief overview of global testing policies for SARS‐CoV‐2

In the so many unknowns within COVID‐19, rapid development of diagnostic assays is a crucial part of the response in this pandemia. In the diagnostic panel, antigens and antibodies should be accurately identified. Testing policies differ as some states, such as the United Kingdom, choose to test only patients who have significant symptoms and are hospitalized. By contrast, countries such as Germany practice testing even for patients with mild symptoms. Such different approaches make a possible comparison of those mortality rates in different countries not always relevant. There are currently no country‐wide protocols for random testing. There have been attempts in Spain, but they have failed due to rapid tests, which have a low degree of accuracy, generating many false‐positive results. In Norway, the issue of random testing has been raised, but it has not been clarified whether people may be forced to accept testing.^109^

On the other hand, the unreasonable use of diagnostic tests for COVID‐19 is potentially harmful and medical errors can occur because no matter how sophisticated a diagnostic test is, it is not perfect. Tests for COVID‐19 do not have a sensitivity (‘true patients’) and specificity (‘true healthy’) of 100%, and therefore, a number of clarifications are required regarding the reasons for testing such as: virus detection, search for the host immune response (ie production of IgM, IgG antibodies), accuracy of the test, testing to identify/diagnose an individual if SARS‐CoV‐2 infection is active, identification of infection early (pre‐/asymptomatic) or late, contagiousness of the case, if the disease is nearing the end and we want to know whether the patient has immunity (the presence of antibodies does not automatically imply immunity).^110^

Viral load may differ from person to person, and in the same person, the kinetics of viral load is variable over time; viral load differs depending on the site of uptake (saliva, pharyngeal exudate, bronchoalveolar lavage).^111^

In general, qualitative serological testing is practised and sometimes it conditions the hospitalization with a negative result. These serological tests can be qualitative, with a Yes/No answer and can be done quickly, or quantitative (ELISA type, in the laboratory)—but anyway their usefulness and meaning are distinct from the usefulness and meaning of viral RNA samples (RT‐PCR). Serological tests, as stated before, measure whether an individual has been previously exposed to the infectious agent,^112^ but do not help to determine the infectivity of the tested person. Clinical evaluation, which considers the probability of infection based on the risk of exposure and the evaluation of clinical signs and symptoms, is crucial in understanding the infectivity of COVID‐19.

## 6 OVERALL CONCLUSIONS

The scientific world should have a realistic approach, this pandemia is not over, and a combination of (immune)therapies/vaccines and standard tests would end it. As the year that is closing gathered so much information, this asset should be thoroughly and wisely used in this year colliding in a global task force to control this infection. Epidemiology of this infection shows that the available estimates of SARS‐CoV‐2 infection prevalence largely depended on the availability of molecular testing and the extent of tested population. Within molecular diagnosis, the viability and infectiousness of the virus in the tested samples should be further investigated.

The fact that SARS‐CoV‐2 has identity with SARS‐CoV and MERS‐CoV proves that SARS‐CoV‐2 is the result of mutations that evolved in a new variant. The immune system participates to the counterattack of the viral infection by pathogen elimination, cellular homeoostasis, tissue repair and generation of memory cells that would be reactivated upon a second encounter with the same virus. In all these stages, we still have knowledge to be gathered regarding antibody persistence, protective effects and immunological memory. Moreover, information regarding the intense pro‐inflammatory action in severe cases still lacks and this is important in stratifying patients for difficult to treat cases.

A holistic approach in this pandemia from human medicine to veterinary medicine, infection tracing, identifying risk factors and predisposition, is needed to develop better prevention and control strategies.

## CONFLICT OF INTEREST

The authors declare that they have no known competing financial interests or personal relationships that could have appeared to influence the work reported in this paper.

## AUTHOR CONTRIBUTIONS

**Monica Neagu:** Conceptualization (equal); formal analysis (equal); resources (equal); supervision (equal); writing‐original draft (equal); writing‐review & editing (equal). **Daniela Calina:** Conceptualization (equal); resources (equal); writing‐original draft (equal); writing‐review & editing (equal). **Anca Oana Docea:** Investigation (equal); Visualization (equal); writing‐original draft (equal); writing‐review & editing (equal). **Carolina Constantin:** Formal analysis (equal); investigation (equal); resources (equal); visualization (equal); writing‐original draft (equal); writing‐review & editing (equal). **Tommaso Filippini:** Data curation (equal); writing‐original draft (equal). **Nikolaos Drakoulis:** Writing‐review & editing (equal). **Marco Vinceti:** Conceptualization (equal); formal analysis (equal); validation (equal); visualization (equal); writing‐original draft (equal); writing‐review & editing (equal). **Konstantinos Poulas:** Writing‐review & editing (equal). **Taxiarchis Konstantinos Nikolouzakis:** Writing‐review & editing (equal). **Demetrios A. Spandidos:** Validation (equal); writing‐original draft (equal); writing‐review & editing (equal). **Aristidis Tsatsakis:** Writing‐review & editing (equal).

## Data Availability

The dataset presented in this study is available from the corresponding author upon reasonable request.
